# Autism Spectrum Disorder Related Functional Connectivity Changes in the Language Network in Children, Adolescents and Adults

**DOI:** 10.3389/fnhum.2017.00418

**Published:** 2017-08-18

**Authors:** Yubu Lee, Bo-yong Park, Oliver James, Seong-Gi Kim, Hyunjin Park

**Affiliations:** ^1^Center for Neuroscience Imaging Research, Institute for Basic Science (IBS) Suwon, South Korea; ^2^Department of Electronic, Electrical and Computer Engineering, Sungkyunkwan University Suwon, South Korea; ^3^Department of Biomedical Engineering, Sungkyunkwan University Suwon, South Korea; ^4^School of Electronic and Electrical Engineering, Sungkyunkwan University Suwon, South Korea

**Keywords:** autism spectrum disorder, language network, resting-state fMRI, graph theoretical analysis, age-related changes

## Abstract

Autism spectrum disorder (ASD) is a neurodevelopmental disability with global implication. Altered brain connectivity in the language network has frequently been reported in ASD patients using task-based functional magnetic resonance imaging (fMRI) compared to typically developing (TD) participants. Most of these studies have focused on a specific age group or mixed age groups with ASD. In the current study, we investigated age-related changes in functional connectivity related measure, degree centrality (DC), in the language network across three age groups with ASD (113 children, 113 adolescents and 103 adults) using resting-state fMRI data collected from the autism brain imaging data exchange repository. We identified regions with significant group-wise differences between ASD and TD groups for three age cohorts using DC based on graph theory. We found that both children and adolescents with ASD showed decreased DC in Broca’s area compared to age-matched TD groups. Adults with ASD showed decreased DC in Wernicke’s area compared to TD adults. We also observed increased DC in the left inferior parietal lobule (IPL) and left middle temporal gyrus (MTG) for children with ASD compared to TD children and for adults with ASD compared to TD adults, respectively. Overall, functional differences occurred in key language processing regions such as the left inferior frontal gyrus (IFG) and superior temporal gyrus (STG) related to language production and comprehension across three age cohorts. We explored correlations between DC values of our findings with autism diagnostic observation schedule (ADOS) scores related to severity of ASD symptoms in the ASD group. We found that DC values of the left IFG demonstrated negative correlations with ADOS scores in children and adolescents with ASD. The left STG showed significant negative correlations with ADOS scores in adults with ASD. These results might shed light on the language network regions that should be further explored for prognosis, diagnosis, and monitoring of ASD in three age groups.

## Introduction

Autism spectrum disorder (ASD) is a neurodevelopmental disability characterized by impairments in language, social interaction and restricted, repetitive, and stereotyped patterns of behavior (American Psychiatric Association, 2000). ASD affects 2% of all children between the ages of 6 and 17 (Blumberg et al., 2013). Deficits in use of language, semantic processing, and interpreting language in context, are universal in ASD patients (Howlin, 2003). Language ability is not only the earliest positive prognostic indicator in children with ASD, but is also closely related to long-term social function. Improved understanding of the brain organization underlying language may shed light on the neural basis of ASD and factors related to clinical severity (Kleinhans et al., 2008). The identification of biomarkers in ASD in language network would be helpful in ensuring an early and accurate diagnosis as well as optimizing effective treatments.

Early task-based functional magnetic resonance imaging (fMRI) studies reported functional connectivity differences of cortical areas separately in children (Wang et al., 2006; Redcay and Courchesne, 2008), adolescents (Knaus et al., 2008), adults (Just et al., 2004; Kana et al., 2006; Gaffrey et al., 2007; Mason et al., 2008; Tesink et al., 2009), or in two age groups (Colich et al., 2012; Williams et al., 2013) on a range of language tasks. Two studies in children with ASD reported that hyper-connectivity in the right inferior frontal gyrus (IFG) during irony processing (Wang et al., 2006) and in right and medial frontal regions during speech perception relative to typically developing (TD) children (Redcay and Courchesne, 2008). Wang et al. (2006) interpreted the hyper-connectivity as the effortful use of normative neural circuitry associated with the processing involved in understanding the mental states. One study reported that adolescents with ASD also showed hyper-connectivity in Broca’s area (left IFG) during semantic integration and word generation task and adolescents with ASD was less lateralized as compared with the TD adolescents (Knaus et al., 2008). Adults with autism demonstrated pattern of relative hyper-connectivity in Wernicke’s area (left superior temporal gyrus (STG) and middle temporal gyrus (MTG)) compared to Broca’s area in sentence comprehension task (Just et al., 2004), semantic decision making task (Harris et al., 2006), and word categorization task (Gaffrey et al., 2007). Two fMRI studies in adults with autism revealed hyper-connectivity in right temporal and right inferior frontal regions in response to increased sentence difficulty or the presence of intentionality (theory-of-mind) information during discourse comprehension (Mason et al., 2008) and in right IFG for processing speaker incongruent sentences (Tesink et al., 2009). The authors reported that these results are consistent with a spillover account, in which the right hemisphere homologs are recruited when the left hemisphere language areas are taxed. One study reported hypo-connectivity within the left hemisphere language network during irony comprehension in children with ASD compared with the TD children, while TD children showed activity in the bilateral language network (Williams et al., 2013). Colich et al. (2012) reported hypo-connectivity in Broca’s area as compared with TD adolescents when viewing visual scenes and making judgments about auditory ironic statements. Many existing fMRI studies of language related areas in different age cohorts with ASD have examined functional connectivity differences using various language tasks. There has been agreement that functional connectivity in each age cohort with ASD is different from TD, but conflicting results have been reported in terms of which brain regions are involved and whether they show hyper- or hypo-connectivity.

Resting-state fMRI (rs-fMRI) has emerged as a powerful tool for examining intrinsic functional brain connectivity without selecting proper tasks for a broad range of ages and clinical groups. The absence of task demands has enabled investigations to examine increasingly earlier stages of development. That is, it allows easier data collection from special populations such as young children with ASD, who have difficulties with long task-based fMRI experiments (Yerys et al., 2009). However, research using task-independent rs-fMRI data to identify and explore the language network in ASD is relatively scarce (Verly et al., 2014). Verly et al. (2014) investigated the functional connectivity of the language network including the cerebellum in children with ASD using rs-fMRI. The study first performed a verb-generation task using task-based fMRI to identify eight joint language components using independent component analysis (ICA), which were then used as seed regions for analysis of the resting-state scan. This study indicated a significantly reduced functional connectivity between IFG and left dorsolateral prefrontal cortex as well as between IFG and right cerebellar cortex children with ASD.

To date, most of these studies have focused on a single age group (e.g., children, adolescents, or adults), mixed age groups, or used a single group of participants spanning a large age range. ASD patients show differential language capabilities as they age from children to adults, and thus neuroimaging assessment of ASD is better performed with these disparate age groups factored in Padmanabhan et al. (2013). Resting-state fMRI studies of the language network in ASD for identifying alterations in functional connectivity in language related regions are scarce across three age cohorts. Little is known about the developmental trajectories of functional connectivity in the language network in ASD. To address these developmental changes in language network using rs-fMRI, functional connectivity needs to be examined in three age cohorts with ASD.

Several fMRI studies found involvement of different networks such as default mode (Mason et al., 2008; Tie et al., 2014; Verly et al., 2014), frontoparietal (Kana et al., 2006; Verly et al., 2014; Nair et al., 2015), and ventral attention (Mason et al., 2008; Shih et al., 2010) networks for language processing. As suggested in the recent literature, classical models of language processing did not fully leverage recent developments in neuroimaging technology and thus studies should consider expanded set of regions (or networks) to better characterize language processing (Fox et al., 2006; Lee et al., 2012; Tie et al., 2014; Verly et al., 2014; Nair et al., 2015; Ardila et al., 2016). A recent study proposed that there were two different language networks in the brain: first, a language reception/understanding system, including a “core Wernicke’s area” involved in word recognition (Brodmann Area (BA) 21, BA 22, BA 41 and BA 42), and a fringe or peripheral area (“extended Wernicke’s area:” BA 20, BA 37, BA 38, BA 39 and BA 40) involved in language associations; second, a language production system (“Broca’s complex”: BA 44, BA 45, and also BA 46, BA 47, parts of BA 6, and the thalamus) from seven studies of fMRI activity during the performance of different language activities (Ardila et al., 2016). Fox et al. (2006) reported that Broca’s and Wernicke’s areas of language network are, to a large extent, the left hemisphere homologs of the right ventral frontal cortex and right temporo-parietal junction (ventral attention network). Tie et al. (2014) reported that frontal component indicated dominant activations in the left frontal and temporal language areas (IFG, middle frontal gyrus (MFG), STG, MTG, and precentral gyrus) and temporal component indicated activations in more extensive regions (left temporal/parietal cortex) for language network using rs-fMRI. Previous studies also reported that language network was related to the ventral attention network and was it had some spatial overlap with ventral attention network which includes the right temporal-parietal junction (supramarginal and superior temporal gyri) and the right ventral frontal cortex (medial and inferior frontal gyri). Thus, we considered many functional networks related to language processing to assess connectivity differences consistent with suggestion of the recent study (Tie et al., 2014; Verly et al., 2014; Nair et al., 2015; Ardila et al., 2016).

In the present study, we investigated age-related changes in functional connectivity related measure, degree centrality (DC), in the language network across three age cohorts (children, adolescents and adults) in ASD and TD groups using rs-fMRI. In order to explore developmental changes in DC, we identified group-wise differences between ASD and TD groups for three age cohorts using DC, which provides information about a node’s centrality or influence within the network based on graph theory (Bullmore and Sporns, 2009). Centrality is a key concept in network analysis and is well-suited to quantify connectivity changes in rs-fMRI whether they are hypo or hyper-connective (Buckner et al., 2009; Bullmore and Sporns, 2009; He et al., 2009). We also investigated general trend of DC values with respect to age cohorts for ASD and TD groups respectively. Our aim was to explore developmental changes in DC for children, adolescents, and adults in language network regions. In addition, we explored if DC values of our findings were related to autism diagnostic observation schedule (ADOS) scores in ASD group. We performed *post hoc* correlation analysis between the results of connectivity analysis and measure of clinical severity such as ADOS communication and ADOS social interaction scores. We hypothesized that DC in the language network using rs-fMRI might demonstrate developmental changes across three age groups with ASD. Particularly, we hypothesized that DC deficit in key language processing regions might play a crucial role for understanding developmental trajectories.

## Materials and Methods

### Participants and Imaging Data

We acquired data from the Autism Brain Imaging Data Exchange I (ABIDE I) and Autism Brain Imaging Data Exchange II (ABIDE II), a publicly available dataset (Di Martino et al., 2014). All data were obtained with informed consent, in accordance with established human participant research procedures. We performed the analyses on 676 participants (329 ASD participants and 347 TD participants) recruited from California Institute of Technology, University of Leuven (Sample 1), New York University (Sample 1 and Sample 2), University of Pittsburgh, University of California Los Angeles, University of Utah, Yale Child Study Center, Georgetown University, Katholieke Universiteit Leuven, Indiana University. To explore the age-related differences in functional connectivity, we divided the data into three age groups of ASD and TD participants: children under 12 years of age (*n* = 240), adolescents from 12 to 19 years of age (*n* = 231), and adults over 19 years of age (*n* = 205). The ASD participants consisted of 113 children, 113 adolescents and 103 adults. The TD participants consisted of 127 children, 118 adolescents and 102 adults. The ASD participants had a clinical DSM-IV diagnosis of Autistic Disorder, Asperger’s syndrome, or Pervasive Developmental Disorder Not-Otherwise Specified (PDD-NOS) using ADOS modules 3 or 4 (Lord et al., 1999) and the Autism Diagnostic Interview–Revised (Lord et al., 1994). The ADOS was not obtained for TD participants. Estimates of intelligence such as FSIQ, VIQ and PIQ were measured using the Wechsler Abbreviated Scale of Intelligence (WASI; Wechsler, 1999) or the Wechsler Intelligence Scale for Children (WISC, Wechsler, 1949). The ADOS has subscores for social interaction (ADOS_SOCIAL) and communication (ADOS_COMM), which are combined into a total score. As shown in Table 1, there were no significant differences (*p* > 0.05) in sex ratios, age, mean framewise displacement (FD; Power et al., 2012), FSIQ, VIQ and PIQ between ASD and TD groups within each of the three age cohorts. Participant demographics and clinical information are provided in Table 1. T1-weighted anatomical data and functional data were acquired using a magnetization-prepared gradient-echo (MPRAGE) sequence and using a gradient echo, echo-planar imaging (EPI) sequence sensitive to blood oxygenation level dependent (BOLD) contrast, respectively. Detailed information on the anatomical and functional imaging parameters used at each site are publicly available on the ABIDE website^1^.

**Table 1:** Demographic information for the ASD and TD participants in the three age groups.

	ASD	TD	*p*-value
**Children** (<12)			
Gender (M : F)	100 : 13	110 : 17	0.66*
Mean age (SD)	9.45 (1.63)	9.66 (1.42)	0.29
Age range	6.05–11.99	6.36–11.95	
Mean FD (SD)	0.05 (0.02)	0.05 (0.02)	0.43
Full scale IQ (SD)	106 (14.70)	108 (10.77)	0.34
Verbal IQ (SD)	105 (13.90)	108 (11.43)	0.15
Performance IQ (SD)	107 (16.27)	106 (12.12)	0.94
ADOS communication	3.24 (1.51)		
ADOS social	7.78 (2.48)		
**Adolescents** (12–19)			
Gender (M : F)	103 : 10	105 : 13	0.58*
Mean age (SD)	15.02 (2.14)	14.68 (2.09)	0.23
Age range	12.03–19.64	12.01–19.8	
Mean FD (SD)	0.04 (0.03)	0.04 (0.01)	0.80
Full scale IQ (SD)	106 (13.66)	108 (11.22)	0.21
Verbal IQ (SD)	106 (13.91)	108 (11.22)	0.22
Performance IQ (SD)	105 (14.55)	107 (12.36)	0.43
ADOS communication	3.46 (1.45)		
ADOS social	7.92 (2.90)		
**Adults** (>20)			
Gender (M : F)	91 : 12	87 : 15	0.52*
Mean age (SD)	26.03 (5.36)	25.47 (4.70)	0.43
Age range	20.0–39.2	20.0–39.39	
Mean FD (SD)	0.04 (0.02)	0.03 (0.02)	0.10
Full scale IQ (SD)	110 (12.87)	112 (8.98)	0.12
Verbal IQ (SD)	110 (14.19)	113 (10.0)	0.12
Performance IQ (SD)	108 (13.62)	109 (9.38)	0.37
ADOS communication	3.58 (1.58)		
ADOS social	7.26 (2.79)		

*Chi-squared test. ASD, autism spectrum disorder; TD, typical development; M, males; F, females; SD, standard deviation; mean FD, mean framewise displacement; IQ, intelligence quotient; ADOS, Autism Diagnostic Observation Schedule.

### Image Preprocessing

We employed the AFNI (Cox, 1996) and FMRIB software library (FSL, Jenkinson et al., 2012) for preprocessing of the T1-weighted anatomical data. The skull tissue was removed using 3dSkullStrip and the magnetic field bias was corrected using FSL’s FAST tool. We processed the rs-fMRI data using FSL software. The preprocessing steps included: (1) removal of the first ten MRI volumes for adjusting the hemodynamic response; (2) realigning data with 6 head motion parameters using MCFLIRT; (3) identifying “bad” time points using a threshold of FD > 0.3 mm and the adjacent (i.e., one preceding and two following) frames (Power et al., 2012); (4) slice timing correction using SLICETIMER; (5) intensity normalization of the fMRI time series data with a value of 10,000; (6) registration of the functional images onto the preprocessed T1-weighted anatomical images and subsequent registration to the Montreal Neurological Institute (MNI) standard space; (7) regressing out the nuisance variables including 24 motion related parameters (Friston et al., 1996), FD of each time point, and signals extracted from white matter and cerebrospinal fluid; and (8) temporal band pass filtering of the voxel time series data retaining frequencies from 0.009 to 0.08 Hz. Finally, we applied spatial smoothing with a 6 mm full-width half-maximum (FWHM) Gaussian blur for all analyses.

### Region of Interest Definition

We defined 64 regions as regions of interest (ROIs) for the functional connectivity analysis of the language network using the Brainnetome Atlas with 246 subregions (210 cortical areas and 36 subcortical regions) of the bilateral hemispheres^2^. The ROIs were defined in the standard MNI space and thus they could be applied to an individual participant’s neuroimaging data which were registered onto the MNI space. The 64 functional ROIs included the bilateral supplementary motor area (LSMA, RSMA: BA 6), bilateral middle frontal gyri (LMFG, RMFG: BA 46), bilateral inferior frontal gyri (LIFG, RIFG: BA 44 and BA 45), bilateral orbital gyri (LOrG, ROrG: BA 47), bilateral superior temporal gyri (LSTG, RSTG: BA 22, BA 38, BA 41 and BA 42), bilateral middle temporal gyri (LMTG, RMTG: BA 21 and BA 37), and bilateral inferior temporal gyri (LITG, RITG: BA 20 and BA 37), and the bilateral inferior parietal lobule (IPL, LIPL, RIPL: BA 39 and BA 40). Those ROIs are involved in multiple functional networks related to language processing including speech comprehension and production and we designated the collection of ROIs as our language network. Our definition of language network contained ROIs of many functional networks and the regions were chosen to explore language related differences. Existing studies also defined language related regions using task fMRI and then explored rs-fMRI properties in the identified regions. We used the same approach except that the regions were derived from the existing studies (Tie et al., 2014; Verly et al., 2014; Nair et al., 2015; Ardila et al., 2016), not from our own task fMRI experiments.

### Construction of the Language Network Using Graph Theory

We constructed the language network using graph theory to analyze functional connectivity among ROIs within the language network. The network consists of a group of nodes connected by edges in a graph structure (He et al., 2007; Bullmore and Sporns, 2009). In our study, we adopted an undirected and unweighted network model. Each ROI used for the language network and the correlation value between two ROIs were represented as node and edge, respectively. Edge values were used as elements of the matrix, which is referred to as the correlation matrix. We first calculated the Pearson correlation coefficient between the mean signal intensity time courses of each ROI pair for the correlation matrix of each participant. To reduce the multisite effect from different data collection sites and potential motion artifacts, we included dummy-coded site variables and mean FD in the regression model for computing the Pearson correlation values used to construct the correlation matrix. A Fisher’s r-to-z transformation was applied to each correlation matrix to obtain an approximately normal distribution of the functional connectivity values. The transformed correlation matrix was stacked. The correlation matrix was binarized by applying a fixed threshold. The threshold was determined from exploring a wide range of sparsity (1~100%). We adopted the minimum value at which all ROIs were connected in the matrices of all participants, which was 19% (Seo et al., 2013).

### Connectivity Analysis

DC, betweenness centrality (BC), and eigenvector centrality (EC) are widely used centrality measures to describe the brain network and can be calculated for each brain region in order to quantify the importance of a given node in terms of network organization. DC is the number of edges connected to a node, which quantifies the information passing through that particular node. BC is the number of shortest paths between any two nodes that run through that node, which represents the information flow of a given node (Rubinov and Sporns, 2010). A node with high DC or BC values is regarded as a hub node which plays an important role in overall network organization (Xia et al., 2014). EC is proportional to the sum of the EC of all nodes directly connected to it. EC is able to capture an aspect of centrality that extends to global features of a given network. DC is one of the most common measures of centrality and it has a straightforward neurobiological interpretation. The nodes with a high DC values are interacting, structurally or functionally, with many other nodes in the network (Rubinov and Sporns, 2010). It is also reported to have higher test-retest reliability than other nodal centrality metrics (Wang et al., 2011; Cao et al., 2014). In our study, we employed DC as the main centrality measure and also used both BC and EC for the additional analysis. We identified regions with significant group-wise differences between ASD and TD participants for all age cohorts (children, adolescents and adults) in terms of DC values as well BC and EC values.

### Correlation of DC with ADOS Scores

We performed *post hoc* correlation analysis between our connectivity findings and ADOS scores related to severity of ASD symptoms in ASD group. Correlation analysis between DC values of identified brain regions and two clinical scores was explored to investigate whether the region with significant differences in functional connectivity was related to the symptoms of autism. DC values of each identified brain region and two ADOS scores were correlated using a general linear model. The significance of the correlation was quantified with *r*- and *p*-value statistics. *P*-values were corrected using the Holm-Bonferroni method (Holm, 1979).

### Statistical Analysis

Statistical differences between ASD and TD groups within each age cohort (children, adolescents, and adults) were assessed using non-parametric permutation tests to avoid the issue of multiple comparisons (Smith et al., 2013). ASD and TD participants were randomly assigned to each age group 5000 times. The null distribution of the DC values was computed from random permutations. Statistically significant differences in DC value were reported if the differences between ASD and TD participants within each of three age groups did not belong to the 95% of the null distribution (determined by two-tailed tests with *p* < 0.05, corrected). The quality of correlation between DC values of each brain region and ADOS scores was quantified using *r*- and *p*-value statistics. We applied the Holm-Bonferroni method to obtain corrected *p*-values.

## Results

### Participants with Head Motion

We excluded participants who had excessive head motion where more than 40% of time points removed during the scrubbing procedure. On this basis, we excluded 18 ASD participants and six TD participants, leaving 329 ASD participants and 347 TD participants for the analysis. The number of participants from each site that were included in our analysis are shown in Supplementary Table S1. To rule out the possibility that differences in head motion between each age cohort in ASD and TD groups could contribute to the results, we calculated the mean value of FD for each participant and compared it between the two age groups. There were no significant differences (*p* > 0.05) in mean FD between each age cohort in ASD and TD groups as shown in Table 1. We compared the distribution of mean FD and percentage of bad time-points between ASD and TD groups and also between each age cohort. The percentage of bad time-points were 0.56% and 0.39% for ASD group and for TD group, respectively. Percentage of bad time-points were 1.2%, 0.37% and 0.18% for children, adolescents, and adults with ASD, respectively. Percentage of bad time-points is 0.72%, 0.35% and 0.13% for TD children, TD adolescents, and TD adults, respectively. The distributions of mean FD and percentage of bad time-points for ASD and TD groups and for each age cohort are plotted in Supplementary Figure S1.

### DC Differences between ASD and TD Groups

**Figure 1:**
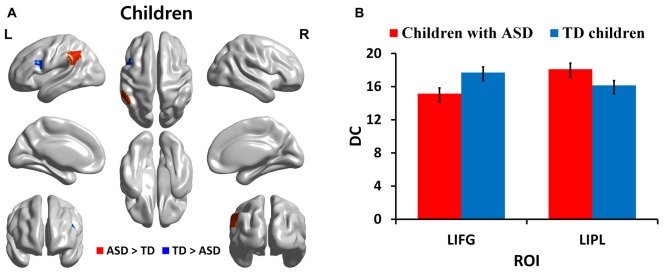
Degree centrality (DC) in regions with significant differences between children with autism spectrum disorder (ASD) and typically developing (TD) children. **(A)** Regions with significant (*p* < 0.05, corrected) difference are represented in red (ASD > TD) and blue (TD > ASD) for children. **(B)** Bar charts show DC values of regions for which there are significant differences in children with ASD and TD children. The ASD group is displayed in red and the TD group in blue with error bar. L, left; IFG, inferior frontal gyrus; IPL: inferior parietal lobule.

**Figure 2:**
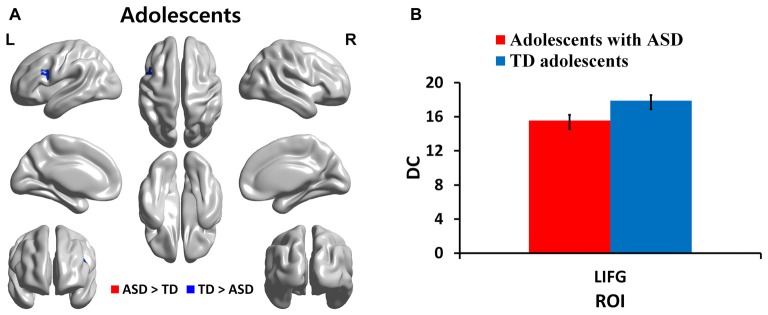
DC in regions with significant differences between adolescents with ASD and TD adolescents. **(A)** Regions with significant (*p* < 0.05, corrected) difference are represented in red (ASD > TD) and blue (TD > ASD) for adolescents. **(B)** Bar charts show DC values of regions for which there are significant differences in adolescents with ASD and TD adolescents. The ASD group is displayed in red and the TD group in blue with error bar. L, left; IFG, inferior frontal gyrus.

**Figure 3:**
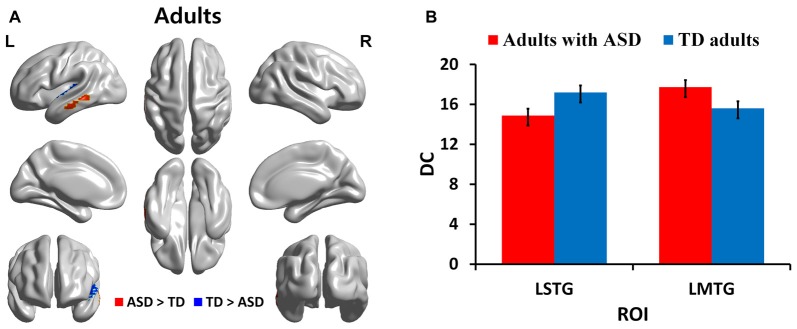
DC in regions with significant differences between adults with ASD and TD adults. **(A)** Regions with significant (*p* < 0.05, corrected) difference are represented in red (ASD > TD) and blue (TD > ASD) for adults. **(B)** Bar charts show DC values of regions for which there are significant differences in adults with ASD and TD adults. The ASD group is displayed in red and the TD group in blue with error bar. L, left; STG, superior temporal gyrus; MTG, middle temporal gyrus.

We identified regions with group-wise differences between ASD and TD participants within each of three age groups using DC values (Table 2). We visualized the regions with significant (*p* < 0.05, corrected) DC difference in red (ASD > TD) and blue (TD > ASD) for each age group (Figures 1A, 2A, 3A). DC value of each region for which there is a significant DC difference was displayed in bar chart with error bar, which is standard error of the mean (Figures 1B, 2B, 3B). Two regions including the left IFG and left IPL exhibited significant (*p* < 0.05, corrected) differences between children with ASD and TD children. Children with ASD showed decreased DC in the left IFG and increased DC in the left IPL relative to TD children (Figure 1B). The left IFG showed significant differences between adolescents with ASD and TD adolescents. Adolescents in ASD exhibited decreased DC in the left IFG compared to TD adolescents (Figure 2B). Comparing between adults with ASD and TD adults, two regions including the left STG and MTG exhibited significant differences. Adults with ASD showed decreased DC in the left STG and increased DC in the left MTG compared to TD adults (Figure 3B). The 64 ROIs selected for the centrality analysis of the language network are shown in Supplementary Figure S2A and *p*-values of regions with the significant group-wise differences between children with ASD and TD children, between adolescents with ASD and TD adolescents and between adults with ASD and TD adults are shown in Supplementary Figures S2B–D, respectively.

**Table 2:** Regions with significant group-wise differences between ASD and TD participants within three age groups using DC.

ASD vs. TD	Significant regions	MNI coordinates			*p*-value, corrected
		*x*	*y*	*z*	
Children	LIFG	−46	13	24	0.0094
	LIPL	−56	−49	38	0.0338
Adolescents	LIFG	−46	13	24	0.0140
Adults	LSTG	−50	−11	1	0.0204
	LMTG	−65	−30	−12	0.0376

*L, left; IFG, inferior frontal gyrus; IPL, inferior parietal lobule; STG, superior temporal gyrus; MTG, middle temporal gyrus*.

### BC and EC Differences between ASD and TD Groups

Additional analysis results for identifying regions with group-wise differences between ASD and TD participants using both BC and EC were reported in the Supplementary Material (Supplementary Tables S2, S3). Using BC, five regions including the bilateral IFG, left orbital gyrus, right STG, and right ITG exhibited significant differences between children with ASD and TD children. The left MFG, right IFG, and right ITG showed significant differences between adolescents with ASD and TD adolescents. There were no regions with significant differences between adults with ASD and TD adults. Using EC, the right ITG showed significant differences between children with ASD and TD children. The left MTG and right ITG showed significant differences between adolescents with ASD and TD adolescents. Comparing between adults with ASD and TD adults, four regions including the left STG, left MTG, right ITG and right IPL exhibited significant differences.

The identified regions using different centrality measures were slightly different. No regions were commonly identified for all three centrality measures. The main centrality measure in this study was DC, so we compared what regions were commonly identified using DC and BC (or DC and EC). The left IFG commonly exhibited significant differences between children with ASD and TD children for both DC and BC. The left STG and MTG showed significant differences between adults with ASD and TD adults for both DC and EC. As shown in Supplementary Figure S3A, children with ASD showed decreased BC in the left IFG relative to TD. Comparing adults with ASD and TD adults, adults with ASD showed decreased EC in the left STG and increased EC in the left MTG compared to TD adults as shown Supplementary Figure S3B. The identified regions showed consistent tendency of increasing or decreasing regardless of the adopted centrality measures (DC, BC and EC) comparing age matched ASD and TD groups.

### Correlation of DC with ADOS Scores

**Figure 4:**
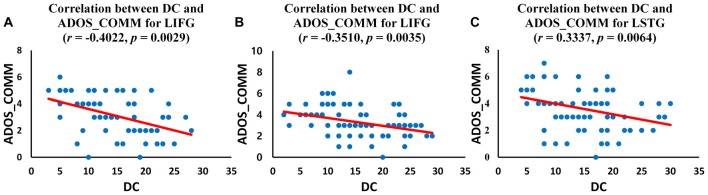
Correlation between autism diagnostic observation schedule (ADOS) scores and DC values of the identified regions for three age cohorts with ASD. **(A)** Correlation between DC value of left inferior frontal gyrus (IFG) and ADOS_COMM score in children with ASD; **(B)** Correlation between DC value of left IFG and ADOS_COMM score in adolescents with ASD; **(C)** Correlation between DC value of left superior temporal gyrus (STG) and ADOS_COMM score in adults with ASD.

We investigated the correlations between ADOS scores and DC values of regions for which the DC was significantly different (*p* < 0.05, corrected) in ASD group. The left IFG demonstrated significant negative correlations with ADOS_COMM score in children with ASD (*r* = −0.4022, *p* = 0.0029; Figure 4A) and in adolescents with ASD (*r* = −0.3510, *p* = 0.0035; Figure 4B). Significant negative correlations were revealed between ADOS_COMM score and the left STG in adults with ASD (*r* = 0.3337, *p* = 0.0064; Figure 4C). Table 3 provides details of the correlation results. We also performed the additional correlations between ADOS scores and BC and EC values of regions for which the BC and EC was significantly different (*p* < 0.05, corrected) in ASD group (Supplementary Tables S4, S5). Significant negative correlation was revealed between ADOS_COMM scores and EC values of the left STG in adults with ASD (*r* = 0.2751, *p* = 0.0465). We found that there were no statistically significant correlations between ADOS scores and BC values of regions for which the BC was significantly different in three age cohorts with ASD.

**Table 3:** Correlation between ADOS scores and DC values of the identified regions.

ASD	ROIs	ADOS_COMM		ADOS_SOCIAL	
		*r*-value	*p*-value, corrected	*r*-value	*p*-value, corrected
Children	***LIFG***	***−0.4022***	***0.0029***	−0.1948	0.1358
	LIPL	0.0013	1	0.0045	1
Adolescents	***LIFG***	***−0.3510***	***0.0035***	−0.2218	0.0525
Adults	***LSTG***	***0.3337***	***0.0064***	−0.1462	0.2075
	LMTG	0.0474	1	−0.0351	0.7634

*ADOS_COMM, autism diagnostic observation schedule communication score; ADOS_SOCIAL, autism diagnostic observation schedule social score. Significant regions and statistical results (*p* < 0.05, corrected) are in bold and italicized*.

## Discussion

In this study, we investigated age-related changes in DC, functional connectivity related measure, in the language network for three age cohorts in ASD and TD groups. We observed decreased DC in the left IFG and increased DC in left IPL in children with ASD compared to TD children. Decreased DC in the left IFG was shown in adolescents with ASD relative to TD adolescents. TD adolescents showed higher DC in left inferior frontal regions compared to adolescents with ASD. Comparing adults in the ASD and TD groups, we found decreased DC in the left STG and increased DC in the left MTG in TD adults compared to adults with ASD. Children and adolescents with ASD showed decreasing DC in left IFG compared to age-matched TD groups. We also investigated correlations of ADOS scores with DC values for identified regions. ADOS_COMM score showed significant (*p* < 0.05, corrected) negative correlations with DC values in the left IFG in children and adolescents with ASD. The left STG demonstrated significant negative correlations with ADOS_COMM score in adults with ASD.

Language processing may be differentially affected in each age group with ASD. To date, fMRI studies of language processing in ASD have examined functional connectivity differences in only one age group such as in children (Wang et al., 2006; Redcay and Courchesne, 2008), adolescents (Knaus et al., 2008), or adults (Just et al., 2004; Kana et al., 2006; Gaffrey et al., 2007; Mason et al., 2008; Tesink et al., 2009), or in two age groups (Colich et al., 2012; Williams et al., 2013) using different language tasks. The existing findings were inconsistent with respect to the regions involved in language processing. Both task-based and resting-state fMRI have been applied to the study of functional connectivity in ASD. It is clear that the methodological choice in both task-based and resting-state approaches can affect outcomes in autism neuroimaging studies (Müller et al., 2011). Connectivity findings derived from rs-fMRI and task fMRI could be divergent for language networks. Additionally, the activation patterns revealed by task-based language fMRI are highly variable across different language tasks (Tie et al., 2014). Despite this issue of difficulty in comparisons between task-based and rs-fMRI functional connectivity, we relate our findings derived from rs-fMRI with existing fMRI findings below.

One study reported that children with ASD had significantly greater activation in the right IFG, left MTG, STG and left postcentral gyrus as compared with TD children during irony processing (Wang et al., 2006). Redcay and Courchesne reported that children with ASD showed greater activation primarily within right and medial frontal regions during speech perception (Redcay and Courchesne, 2008). These studies in children with ASD showed significantly increased connectivity in right language areas. Williams et al. (2013) reported that the children with ASD had lower activation within the left hemisphere language network during irony comprehension as compared with the TD children, while TD children showed activity in the bilateral inferior frontal gyri. Lai et al. (2012) observed decreased connectivity in left IFG in children with ASD relative to TD children during speech stimulation. Our finding of decreased DC in Broca’s area (left IFG) in children with ASD compared to TD children is consistent with the previous studies.

Comparing adolescents in the ASD and TD groups, our results showed a decreased DC in Broca’s area in adolescents with ASD. Knaus et al. (2008) reported increased activation in the right IFG, MFG, MTG, precentral gyrus, orbito-frontal gyrus, and superior parietal gyrus in adolescents with ASD during a response naming task. Two studies reported decreased connectivity in Broca’s area in adolescents with ASD during semantic integration and word-generation task (Knaus et al., 2008) and viewing visual scenes and making judgments about auditory ironic statements (Colich et al., 2012). Our results showed decreased DC in the left IFG compared to TD adolescents. The previous studies are consistent with our study. Taken together, we found significantly decreased DC in the left IFG in both children and adolescents with ASD compared to age-matched TD groups. Some authors reported that decreased connectivity in left IFG during language tasks might be associated with language specific deficits that could result from upstream impairments in perceptual or linguistic processing of speech stimuli in ASD (Müller, 2007; Groen et al., 2008).

Several studies in adults with ASD have demonstrated differences in Wernicke’s area (left MTG and STG) during semantic tasks, with some studies reporting increased activation of Wernicke’s area in adults with ASD (Just et al., 2004; Harris et al., 2006; Gaffrey et al., 2007). Wernicke’s area has traditionally been defined as the posterior STG and accepted as playing a pivotal role in word comprehension (Ross, 2010). Later, the left posterior superior temporal sulcus (Price, 2000), MTG, angular gyrus, and the supramarginal gyrus were also defined as parts of Wernicke’s area (Bogen and Bogen, 1976; Rauschecker and Scott, 2009). Our finding showed that adults with ASD had decreased DC in the left STG and increased DC in the left MTG compared to TD adults. Our finding of increased DC in Wernicke’s area (left MTG) in adults with ASD is partly consistent with the previous studies.

In our study, we explored if our findings of altered DC in the language network were related to ADOS scores, which is a diagnostic tool that measures clinical severity of ASD. In the current study, notable results were that the ADOS_COMM score was negatively correlated with DC values in the left IFG in children and adolescents with ASD. Also of interest was that the ADOS_COMM score was negatively correlated with the left STG in adults with ASD. Several rs-fMRI studies have found significant relationship between functional connectivity measures and autism characteristics measured by the ADOS scores (Monk et al., 2009; Assaf et al., 2010; Dinstein et al., 2011; Nair et al., 2013; Redcay et al., 2013). Dinstein et al. (2011) reported higher ADOS_COMM score with decreased connectivity in the IFG. Similarly ADOS_COMM scores were reported to be negatively correlated with the right lateral parietal to anterior medial prefrontal cortex connectivity by Redcay et al. (2013). ADOS_COMM and ADOS_SOCIAL scores were also reported to be negatively correlated with right motor cortex to thalamus connectivity (Nair et al., 2013) and with connectivity *z*-scores of precuneus (Assaf et al., 2010). Monk et al. (2009) reported that higher ADOS_SOCIAL score correlated with decreased posterior cingulate cortex to right supplementary motor area connectivity. They also reported that severe ASD symptom was correlated with increased posterior cingulate cortex to right posterior parahippocampal gyrus connectivity (Monk et al., 2009). Many of the commonly reported regions with decreased functional connectivity in ASD group are known to be involved in the relevant behavioral capacities. The degree to which these altered connectivities are predictive and specific for the altered behaviors is unknown (Rane et al., 2015). In our study, a significant negative relationship between ADOS_COMM score and DC value in the two regions might account for the greater impairment related to decreased DC in these regions. Our results can therefore be considered reinforcing, as neuroimaging analyses were closely linked with ADOS, a known indicator of ASD severity.

Our study has some limitations. First, our definition of language related ROIs came from many functional networks. Thus, the chosen ROIs do reflect language processing regions but also are related to other functions as well. A smaller set of ROIs more specific to language processing would potentially lead to more sensitive results. Second, our study is a cross-sectional rather than a longitudinal study, and thus the conclusions that can be drawn regarding the developmental process in ASD are limited. To validate this issue, we need to study connectivity differences in longitudinal data, which is left for future studies. Still, the age-related changes of the regions identified in the current study could be helpful for future studies tracking developmental stages of functional connectivity in the language network in ASD.

## Conclusion

In summary, the current study investigated differences in DC of language network between ASD and TD groups across three age cohorts using rs-fMRI. We found that both children and adolescents with ASD showed decreased DC in Broca’s area compared to age-matched TD groups. Adults with ASD showed decreased DC in Wernicke’s area (left STG), whereas adults with ASD showed increased DC in the left MTG compared to TD adults. We also observed increased DC in the left IPL in children with ASD compared to TD children. Furthermore, the DC values of left IFG in the language network demonstrated negative correlations with ADOS scores related to autism severity in children and adolescents with ASD. The left STG demonstrated significant negative correlations with ADOS score in adults with ASD. Overall, functional differences occurred in key language processing regions such as Broca’s area and Wernicke’s area related to language production and comprehension across three age cohorts. We believe the age-related changes of the regions identified in the current study could be helpful for future studies considering developmental stage of functional connectivity in the language network in ASD.

## Ethics Statement

The Institutional Review Board (IRB) of Sungkyunkwan University approved our retrospective study. All subjects gave written informed consent in accordance with the Declaration of Helsinki. The protocol was approved by the IRB of Sungkyunkwan University.

## Author Contributions

YL, S-GK and HP wrote the manuscript and B-yP and OJ aided the experiments. HP is the guarantor of this work and, as such, had full access to all the data in the study and takes responsibility for the integrity of the data and the accuracy of the data analysis.

## Conflict of Interest Statement

The authors declare that the research was conducted in the absence of any commercial or financial relationships that could be construed as a potential conflict of interest. The reviewer H-YL and handling Editor declared their shared affiliation, and the handling Editor states that the process nevertheless met the standards of a fair and objective review.

## Footnotes

^1^http://fcon_1000.projects.nitrc.org/indi/abide/

^2^http://atlas.brainnetome.org
