# Supplementary material for: Autism Spectrum Disorder Related Functional Connectivity Changes in the Language Network in Children, Adolescents and Adults
Source: Front Hum Neurosci. 2017 Aug 18;11:418. doi: 10.3389/fnhum.2017.00418 (PMC5563353; doi:10.3389/fnhum.2017.00418)
Supplement: Supplementary file 1 [file Data_Sheet_1.doc]

Supplementary Material

# Autism spectrum disorder related functional connectivity changes in the language network in children, adolescents, and adults

Yubu Lee^1^, Bo-yong Park^1,2^, Oliver James^1^, Seong-Gi Kim^1,3^ , Hyunjin Park^1,4*^

*** Correspondence:** Hyunjin Park: hyunjinp@skku.edu

Supplementary Figures and Tables

## Supplementary Figures


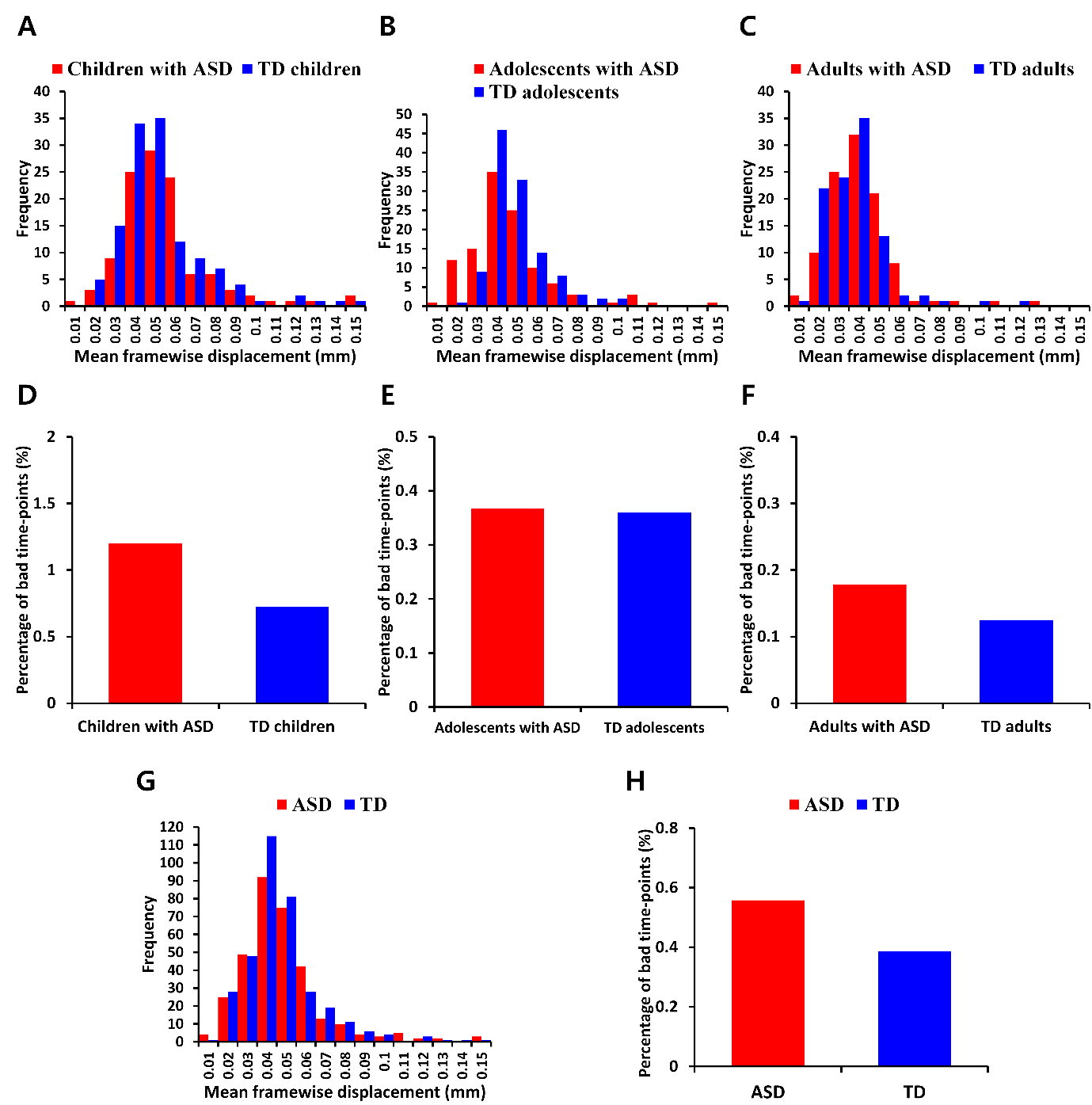


**Supplementary Figure 1.** Distribution of mean FD and percentage of bad time-points for ASD and TD groups and for each age groups. (A) Distribution of individual mean FD for children with ASD and TD children. (B) Distribution of individual mean FD for adolescent with ASD and TD adolescents. (C) Distribution of individual mean FD for adults with ASD and TD adults. (D) Percentage of bad time-points for children with ASD and TD children. (E) Percentage of bad time-points for adolescent with ASD and TD adolescents. (F) Percentage of bad time-points for adults with ASD and TD adults. (G) Distribution of mean FD for ASD and TD groups. (H) Percentage of bad time-points for ASD and TD groups. Mean FD (mm) is shown on the *x*-axis and frequency of mean FD is shown the *y*-axis. The ASD group is displayed in red and the TD group in blue.


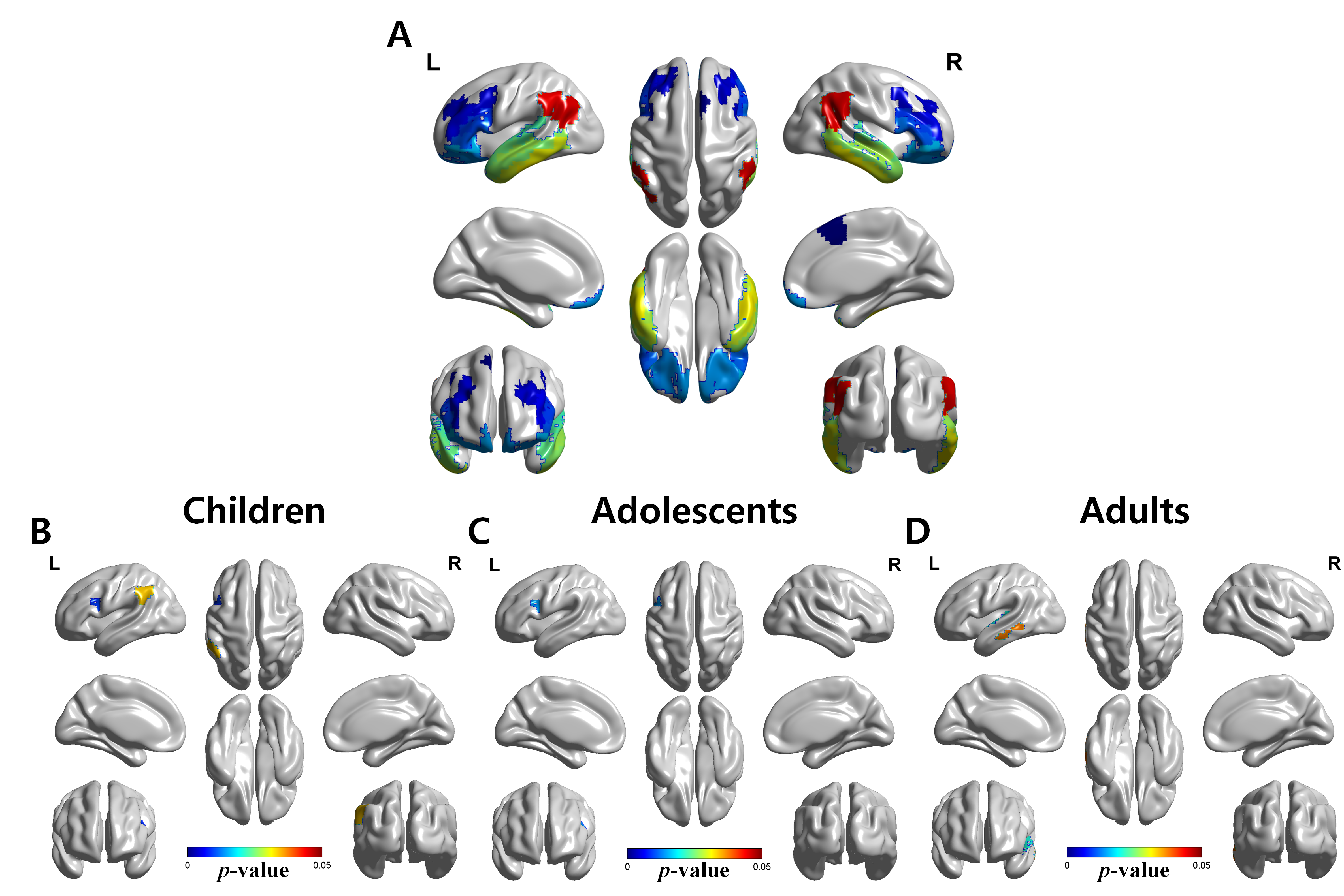


**Supplementary Figure 2.** Regions with group-wise differences between ASD and TD participants within each of three age groups using degree centrality values. (A) 64 ROIs selected for the functional connectivity analysis of the language network. (B), (C), and (D) display regions with the significant group-wise differences (*p* < 0.05, corrected) between children with ASD and TD children, between adolescents with ASD and TD adolescents, and between adults with ASD and TD adults, respectively.


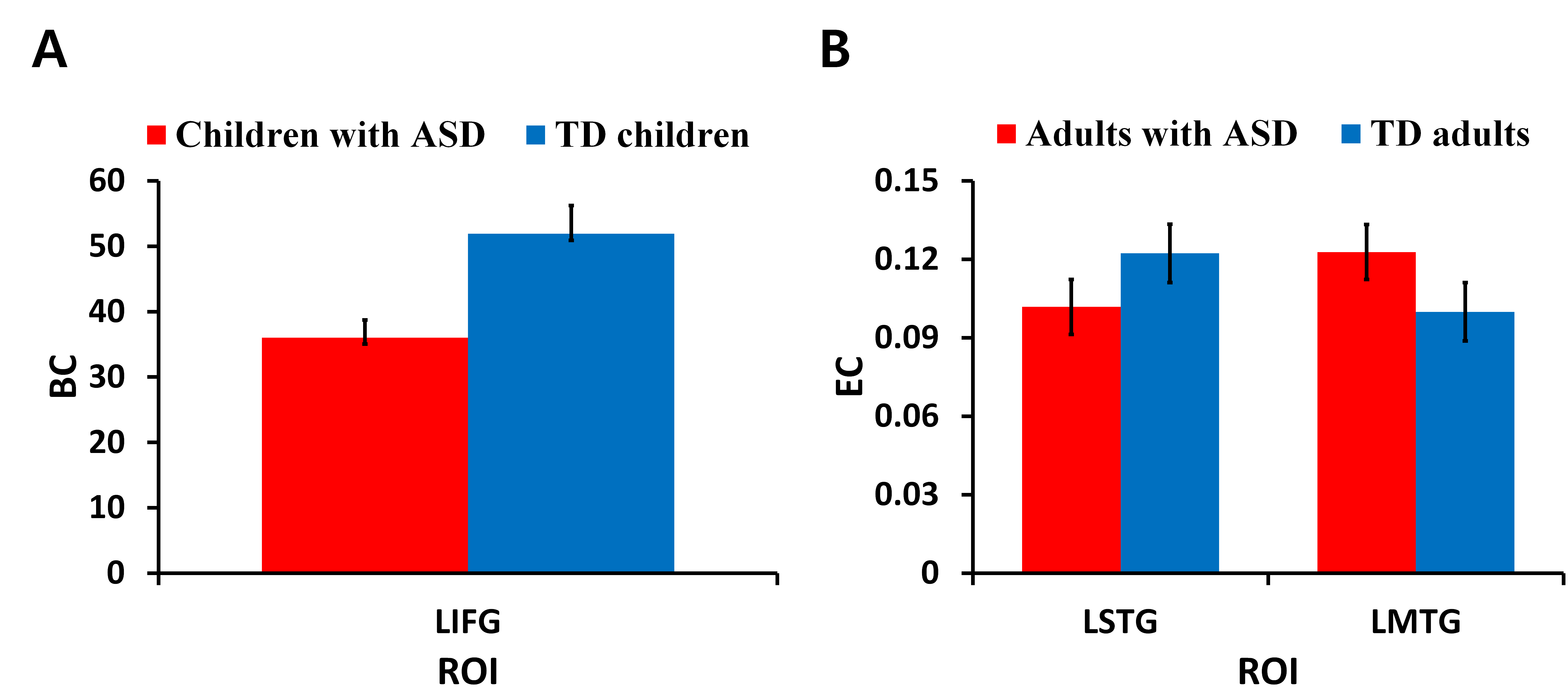


**Supplementary Figure 3.** (A) Regions with significant centrality differences between children with ASD and TD children using betweenness centrality (BC). (B) Regions with significant centrality differences between adults with ASD and TD adults using eigenvector centrality (EC). Bar charts show centrality values of regions for which there were significant differences in ASD and TD groups. The ASD group is displayed in red and the TD group in blue with error bar. L, left; IFG, inferior frontal gyrus; STG, superior temporal gyrus; MTG, middle temporal gyrus.

Supplementary Tables

**Supplementary Table 1.** The number of datasets from each site included in our analysis.

| **Site** | | **ASD** | | | **TD** | | |
| --- | --- | --- | --- | --- | --- | --- | --- |
|  |  | Children  (n=113) | Adolescents  (n=113) | Adults  (n=103) | Children  (n=127) | Adolescents  (n=118) | Adults  (n=102) |
| ABIDE  I | CALTECH |  |  | 14 |  |  | 12 |
|  | LEUVEN_1 |  |  | 7 |  |  | 14 |
|  | NYU | 34 | 21 | 16 | 33 | 45 | 27 |
|  | PITT | 2 | 13 | 9 |  | 15 | 9 |
|  | UCLA | 15 | 27 |  | 14 | 30 |  |
|  | USM |  |  | 20 |  | 11 | 14 |
|  | YALE | 12 | 15 |  | 11 |  |  |
| ABIDE II | GU | 10 | 8 |  | 36 | 11 |  |
|  | IU |  | 7 | 10 |  |  | 14 |
|  | KUL |  | 6 | 20 |  |  |  |
|  | NYU_1 | 20 | 6 | 3 | 23 |  |  |
|  | NYU_2 | 17 |  |  |  |  |  |
|  | UCLA | 3 | 7 |  | 10 | 3 |  |
|  | USM |  | 3 | 4 |  | 3 | 12 |

ABIDE I, Autism Brain Imaging Data Exchange I; ABIDE II, Autism Brain Imaging Data Exchange II; CALTECH, California Institute of Technology; LEUVEN_1, University of Leuven (Sample 1); NYU, New York University; PITT, University of Pittsburgh; UCLA, University of California Los Angeles; USM, University of Utah; YALE, Yale Child Study Center; GU, Georgetown University; IU, Indiana University; KUL, Katholieke Universiteit Leuven; NYU_1, New York University (Sample 1); NYU_2, New York University (Sample 2).

**Supplementary Table 2.** Regions with significant group-wise differences between ASD and TD participants within three age groups using BC.

| **ASD vs. TD** | **Significant regions** | **MNI coordinates** | | | ***p*-value, corrected** |
| --- | --- | --- | --- | --- | --- |
|  |  | **x** | **y** | **z** |  |
| **Children** | LIFG | -52 | 13 | 6 | 0.0020 |
|  | RIFG | 54 | 14 | 11 | 0.0352 |
|  | LOrG | -23 | 38 | -18 | 0.0096 |
|  | RSTG | 51 | -4 | -1 | 0.0288 |
|  | RITG | 53 | -52 | -18 | 0.2320 |
| **Adolescents** | LMFG | -27 | 43 | 31 | 0.0370 |
|  | RIFG | 54 | 14 | 11 | 0.0042 |
|  | RITG | 53 | -52 | -18 | 0.0152 |
| **Adults** | *none* | - | - | - | - |

L, left; R, right; IFG, inferior frontal gyrus; OrG: orbital gyrus; STG, superior temporal gyrus; ITG, inferior temporal gyrus; MFG, middle frontal gyrus.

**Supplementary Table 3.** Regions with significant group-wise differences between ASD and TD participants within three age groups using EC.

| **ASD vs. TD** | **Significant regions** | **MNI coordinates** | | | ***p*-value, corrected** |
| --- | --- | --- | --- | --- | --- |
|  |  | **x** | **y** | **z** |  |
| **Children** | RITG | 55 | -11 | -32 | 0.0092 |
| **Adolescents** | LMTG | -59 | -58 | 4 | 0.0396 |
|  | RITG | 55 | -11 | -32 | 0.0208 |
| **Adults** | LSTG | -50 | -11 | 1 | 0.0292 |
|  | LMTG | -65 | -30 | -12 | 0.0180 |
|  | RITG | 55 | -11 | -32 | 0.0204 |
|  | RIPL | 53 | -54 | 25 | 0.0192 |

L, left; R, right; ITG, inferior temporal gyrus; MTG, middle temporal gyrus; STG, superior temporal gyrus; IPL, inferior parietal lobule.

**Supplementary Table 4.** Correlation between ADOS scores and BC values of the identified regions.

| **ASD** | **ROIs** | **ADOS_COMM** | | **ADOS_SOCIAL** | |
| --- | --- | --- | --- | --- | --- |
|  |  | *r*-value | *p*-value, corrected | *r*-value | *p*-value, corrected |
| **Children** | LIFG | -0.1212 | 1 | -0.0217 | 0.86926 |
|  | RIFG | 0.2600 | 0.2233 | 0.0290 | 1 |
|  | LOrG | 0.0077 | 0.9537 | 0.1152 | 1 |
|  | RSTG | -0.1268 | 1 | -0.0490 | 1 |
|  | RITG | 0.0891 | 0.9968 | 0.0924 | 1 |
| **Adolescents** | LMFG | -0.1205 | 0.8899 | -0.1695 | 0.4219 |
|  | RIFG | 0.0346 | 0.7651 | 0.0418 | 1 |
|  | RITG | -0.0421 | 1 | -0.0199 | 0.8634 |
| **Adults** | none | *-* | *-* | *-* | *-* |

ADOS_COMM, autism diagnostic observation schedule communication score; ADOS_SOCIAL, autism diagnostic observation schedule social score.

**Supplementary Table 5.** Correlation between ADOS scores and EC values of the identified regions.

| **ASD** | **ROIs** | **ADOS_COMM** | | **ADOS_SOCIAL** | |
| --- | --- | --- | --- | --- | --- |
|  |  | *r*-value | *p*-value, corrected | *r*-value | *p*-value, corrected |
| **Children** | RITG | -0.1167 | 0.3744 | 0.0527 | 0.6891 |
| **Adolescents** | LMTG | -0.1602 | 0.3281 | -0.0488 | 0.6736 |
|  | RITG | 0.0514 | 0.6569 | 0.0510 | 1 |
| **Adults** | ***LSTG*** | ***-0.2751*** | ***0.0465*** | -0.1526 | 0.7529 |
|  | LMTG | 0.0406 | 0.7276 | -0.0441 | 1 |
|  | RITG | 0.1305 | 0.5225 | 0.0063 | 0.9569 |
|  | RIPL | 0.2298 | 0.1376 | 0.1234 | 0.8650 |

ADOS_COMM, autism diagnostic observation schedule communication score; ADOS_SOCIAL, autism diagnostic observation schedule social score. Significant regions and statistical results (*p* < 0.05, corrected) are in bold and italicized.
